# Two new *Colletotrichum* species from *Michelia × alba* and *Myristica fragrans* in Hainan Province, China

**DOI:** 10.3897/mycokeys.141.203785

**Published:** 2026-09-30

**Authors:** Zixv Dong, Yibo Yuan, Yan Lv, Zhaoxue Zhang, Xiuguo Zhang, Jiwen Xia

**Affiliations:** 1 College of Agriculture and Forestry, Linyi University, Linyi, Shandong, 276000, China College of Agriculture and Forestry, Linyi University Linyi China; 2 College of Plant Protection, Shandong Agricultural University, Taian, Shandong, 271018, China College of Plant Protection, Shandong Agricultural University Taian China; 3 Agriculture and Rural Service Center of Jiuqu Street, Hedong District, Linyi, Shandong, 276000, China Agriculture and Rural Service Center of Jiuqu Street, Hedong District Linyi China

**Keywords:** *
Glomerellales
*, multi-gene phylogeny, new taxa, systematics, taxonomy

## Abstract

*Colletotrichum*‌ species are distributed worldwide and have been reported as plant pathogens, saprophytes, endophytes, human pathogens, and insect pathogens. In this study, four strains of *Colletotrichum* were isolated from symptomatic leaves of *Michelia × alba* and *Myristica fragrans*, which were collected in Hainan Province, China. Based on morphological examination and multilocus phylogenetic analyses of a combined ACT, CHS-1, GAPDH, HIS3, ITS, and TUB2 dataset, two new species were identified in the *C. gigasporum* species complex and are described herein as *C. micheliae* and *C. myristicae***spp. nov**. These new species were compared with other *Colletotrichum* species, and comprehensive descriptions and micrographs are provided.

## Introduction

*Colletotrichum* was introduced by Corda (1831) and is currently recognized as the only genus in *Glomerellaceae*, *Glomerellales*, *Sordariomycetes*, *Ascomycota* (Liu et al. 2022). Von Arx (1957) revised the genus, reducing approximately 750 species to 11 taxa based on morphological characters. Sutton (1980) accepted 22 species, while Sutton (1992) accepted 39 species based on morphological and cultural characteristics. Cannon et al. (2000) accepted approximately 40 species based on morphological, cultural, and pathogenicity studies. Hyde et al. (2009) provided the first comprehensive overview of this genus, recognizing 66 commonly used names and 19 doubtful names, while also emphasizing the need for a molecular-based revision of the genus. Subsequently, many researchers studied the genus based on morphological and molecular phylogenetic analyses (Damm et al. 2012a, 2012b; Weir et al. 2012; Liu et al. 2015). Liu et al. (2022) accepted 280 species with available molecular data, updated the species diversity of *Colletotrichum*, and provided a phylogenomic overview. However, recent taxonomic studies and the EPPO-Q-BANK database suggest that there are currently approximately 334 accepted species (https://qbank.eppo.int/, accessed June 2026). *Colletotrichum* species are distributed across 15 species complexes: acutatum, agaves, boninense, caudatum, dematium, destructivum, dracaenophilum, gigasporum, gloeosporioides, graminicola, magnum, orbiculare, orchidearum, spaethianum, and truncatum, as well as a number of singletons (Liu et al. 2022).

*Colletotrichum* is a globally distributed, species-rich genus that is known as the causal agent of anthracnose diseases across nearly every major agricultural, horticultural, and forestry crop system (Guevara-Suarez et al. 2022; Liu et al. 2022; Zhang et al. 2024; Zhang and Fan 2025). With an extensive range of hosts in different plant families and species, this genus causes severe pre- and post-harvest yield losses, quality degradation, and economic damage valued at billions of dollars annually (Dean et al. 2012). The genus has undergone numerous revisions in recent decades with the addition, typification, and synonymy of many species (Jayawardena et al. 2016; Liu et al. 2022). The taxonomy of *Colletotrichum* has shifted from morphology-based identification to a robust polyphasic approach integrating multilocus phylogenetics, phylogenomics, and morphological, geographical, and ecological data. Phylogenetic analysis based on concatenated loci and the application of Genealogical Concordance Phylogenetic Species Recognition (GCPSR) have proven to be powerful tools for species delimitation within the genus *Colletotrichum* (Cannon et al. 2012; Jayawardena et al. 2016; Bhunjun et al. 2021; Liu et al. 2022; Norphanphoun et al. 2025).

The *Colletotrichum gigasporum* species complex was first described by Liu et al. (2014), including six species, i.e., *C. arxii*, *C. gigasporum*, *C. magnisporum*, *C. pseudomajus*, *C. radicis*, and *C. vietnamense*. Subsequently, Silva et al. (2018) described *C. serranegrense* as an endophyte from a root of the endangered orchid *Cattleya jongheana* (*Orchidaceae*) in Brazil. Zhou et al. (2019) described *C. jishouense* from healthy roots and stems of *Nothapodytes pittosporoides* (*Icacinaceae*) as an endophyte in China. Liu et al. (2022) described *C. subvariabile* and *C. variabile* from healthy leaves of an unknown plant, and *C. zhaoqingense* from *Carica papaya*, a dead petiole of a palm, and *Musa* sp. in China. Jayawardena et al. (2022) described *C. flexuosum* from leaf spots of *Xanthophyllum sylvestre* in Vietnam. Armand et al. (2023) described *C. durionigenum* from rotting durian fruit in Thailand. Takata et al. (2024) described *C. kapreanum* from a wilted cacao cherelle in the Philippines. Although *C. gigasporum* is the most common species, other species in the *C. gigasporum* species complex have seldom been reported, making this complex the least common in the genus (Talhinhas and Baroncelli 2021).

Hainan is one of the key global hotspots for biodiversity conservation and also one of the regions in China with the richest biodiversity, characterized by a unique tropical rainforest ecosystem, along with typical marine ecosystems such as coral reefs, mangroves, and seagrass beds. The annual mean temperature is 22–27 °C, and the annual precipitation is 1000–2600 mm. Fungal diversity in Hainan is high, and many microfungi have been discovered there (Ai et al. 2025; Li et al. 2025; Liu et al. 2025; Zhang et al. 2025).

In the present study, mycological surveys of plant leaves were conducted in Hainan, China. Through a combination of detailed morphological examination and a comprehensive six-locus phylogenetic analysis (ACT, CHS-1, GAPDH, HIS3, ITS, and TUB2), this study aims to characterize new species of *Colletotrichum* and expand knowledge of fungal diversity in Hainan, China.

## Materials and methods

### Sample collection, isolation, and morphology

From 2023 to 2024, a large number of plant leaves that exhibited distinct signs of fungal necrosis or typical spot symptoms were collected from gardens, streets, or the wild in or near the Hainan Tropical Botanical Garden and Wuzhishan National Nature Reserve in Hainan Province, China. Pure colonies were obtained using tissue isolation techniques (Li et al. 2025). Fragments (25 mm^2^) were cut from the edges of diseased tissues, immersed in 75% ethanol solution for 1 min, and rinsed in sterile water for 30 s, then immersed in 10% sodium hypochlorite solution for 1 min and rinsed three times in sterile water for 30 s. Fragments were blotted dry on sterilized filter paper and placed on potato dextrose agar (PDA; 200 g potato, 20 g dextrose, 20 g agar, and deionized water to 1000 mL; natural pH) and incubated at 25 °C for 3 days. The hyphae were picked from the periphery of the PDA colonies and inoculated onto new PDA plates to obtain pure cultures (Li et al. 2025). The strains were preserved in 10% sterilized glycerol and stored at 4 °C for future detailed studies. Dried cultures were deposited in the Herbarium of the Department of Plant Pathology, Shandong Agricultural University, Taian, China (HSAUP). The living cultures were deposited in the Shandong Agricultural University Culture Collection (SAUCC) and the China General Microbiological Culture Collection Center (CGMCC).

The cultural characteristics of the colonies were observed and photographed using a Sony Alpha 6400L digital camera (Sony Group Corporation, Tokyo, Japan) after 7 and 14 days of incubation at 25 °C in darkness. The micromorphological characteristics of the colonies were observed with an Olympus SZX10 stereomicroscope and an Olympus BX53 microscope (Olympus Corporation, Tokyo, Japan), along with a BioHD-A20c color digital camera (FluoCa Scientific, Shanghai, China). Structural measurements were carried out using Digimizer software (v. 5.6.0), with a minimum of 20 measurements taken for each structure, such as conidiophores, conidiogenous cells, and conidia. The taxonomic information of the new taxa was submitted to MycoBank (http://www.mycobank.org).

### Phylogenetic analyses

The genomic DNA of the fungi was extracted using a modified cetyltrimethylammonium bromide (CTAB) method (Wang et al. 2023; Li et al. 2025). Six loci were amplified and sequenced: partial actin (ACT), chitin synthase 1 (CHS-1), glyceraldehyde-3-phosphate dehydrogenase (GAPDH), histone 3 (HIS3), internal transcribed spacer (ITS), and beta-tubulin 2 (TUB2). The primers used for polymerase chain reaction (PCR) were ACT-512F/ACT-783R for ACT (Carbone and Kohn 1999), CHS-79F/CHS-345R for CHS-1 (Carbone and Kohn 1999), GDR1/GDF1 for GAPDH (Guerber et al. 2003), CYLH3F/CYLH3R for HIS3 (Crous et al. 2004), ITS1/ITS4 for ITS (White et al. 1990), and T1/Bt2b for TUB2 (Glass and Donaldson 1995). PCR was performed in a 25 μL reaction volume consisting of 12.5 μL of 2 × Hieff Canace® Plus PCR Master Mix (Cat. No. 10154ES03, Yeasen Biotechnology, Shanghai, China), 1 μL each of the forward and reverse primers (TsingKe, Qingdao, China), 1 μL of template genomic DNA, and 9.5 μL of double-distilled water. The amplification programs followed the thermal cycling conditions described by Damm et al. (2009). PCR products were separated using a 1% agarose gel and Safe Red (RM02852 and RM19009, ABclonal, Wuhan, China), and the fragments were visualized under UV light. Gel fragments were extracted using a Gel Extraction Kit (Cat. No. AE0101-C, Shandong Sparkjade Biotechnology Co., Ltd., Jinan, China). The purified PCR products were subjected to bidirectional sequencing by Sangon Biotech Company Limited (Shanghai, China). All sequences generated in this study were deposited in GenBank under the accession numbers provided in Table 1.

**Table 1.** GenBank accession numbers used in the phylogenetic analyses.

**Species**	**Strain**	**Host/substrate**	**Locality**	**GenBank accession numbers**					
				**ACT**	**CHS-1**	**GAPDH**	**HIS3**	**ITS**	**TUB2**
*Colletotrichum arxii*	CBS 169.59	*Oncidium excavatum*	Netherlands	KF687784	KF687781	KF687824	KF687846	KF687717	KF687868
	CBS 132511^*^	*Paphiopedilum* sp.	Germany	KF687802	KF687780	KF687843	KF687858	KF687716	KF687881
*Colletotrichum durionigenum*	MFLUCC 22-0111^*^	durian rotting fruit	Thailand	OP744503	OP744504	OP744505	NA	OP740244	OP744506
*Colletotrichum flexuosum*	CBS 134419^*^	*Xanthophyllum sylvestre*	Vietnam	MZ444586	MZ444588	MZ444584	MZ444590	MZ444580	MZ444582
	CBS 137338	*Xanthophyllum sylvestre*	Vietnam	MZ444587	MZ444589	MZ444585	MZ444591	MZ444581	MZ444583
*Colletotrichum gigasporum*	CBS 101881	*Solanum betaceum*	New Zealand	KF687797	KF687777	KF687841	KF687861	KF687736	KF687886
	CBS 125387	*Theobroma cacao*	Panama	KF687788	KF687765	KF687834	KF687851	KF687733	KF687873
	CBS 125476	*Coffea* sp.	Vietnam	KF687790	KF687767	KF687833	KF687853	KF687728	KF687875
	CBS 133266^*^	*Centella asiatica*	Madagascar	NA	KF687761	KF687822	KF687844	KF687715	KF687866
*Colletotrichum gloeosporioides*	CBS 953.97^*^	*Citrus sinensis*	Italy	GQ856782	GQ856733	GQ856762	NA	GQ485605	GQ849434
*Colletotrichum jishouense*	GZU_HJ2_G2	*Nothapodytes pittosporoides*	China	MH708134	NA	MH681657	NA	MH482931	MH727472
	GZU_HJ2_G3^*^	*Nothapodytes pittosporoides*	China	MH708135	NA	MH681658	NA	MH482929	MH727473
	GZU_HJ2_G4	*Nothapodytes pittosporoides*	China	MH708136	NA	MH681659	NA	MH482932	MH727474
	GZU_HJ3_J5	*Nothapodytes pittosporoides*	China	MH708137	NA	MH492706	NA	MH482930	NA
*Colletotrichum kapreanum*	PH22T135^*^	*Theobroma cacao*, cherelle	Philippines	LC750350	LC750349	LC750348	NA	LC750347	LC750351
*Colletotrichum magnisporum*	CBS 398.84^*^	unknown	unknown	KF687803	KF687782	KF687842	KF687865	KF687718	KF687882
***Colletotrichum micheliae***	SAUCC 6771-5^*^	***Michelia × alba***	**China**	**PZ489857**	**PZ489861**	**PZ489865**	**PZ489869**	**PZ490883**	**PZ489873**
	**SAUCC 6771-6**	***Michelia × alba***	**China**	**PZ489858**	**PZ489862**	**PZ489866**	**PZ489870**	**PZ490884**	**PZ489874**
***Colletotrichum myristicae***	SAUCC 8077-1^*^	***Myristica fragrans***	**China**	**PZ489859**	**PZ489863**	**PZ489867**	**PZ489871**	**PZ490885**	**PZ489875**
	**SAUCC 8077-2**	***Myristica fragrans***	**China**	**PZ489860**	**PZ489864**	**PZ489868**	**PZ489872**	**PZ490886**	**PZ489876**
*Colletotrichum pseudomajus*	CBS 571.88^*^	*Camellia sinensis*	China	KF687801	KF687779	KF687826	KF687864	KF687722	KF687883
*Colletotrichum radicis*	CBS 529.93^*^	unknown	Costa Rica	KF687785	KF687762	KF687825	KF687847	KF687719	KF687869
*Colletotrichum serranegrense*	COAD 2100^*^	*Cattleya jongheana*	Brazil	KY407892	KY407894	NA	NA	KY400111	KY407896
	COAD 2105	*Cattleya jongheana*	Brazil	KY407893	KY407895	NA	NA	KY400112	KY407897
*Colletotrichum* sp.	CBS 159.50	*Derris* sp.	Indonesia	KF687800	KF687778	KF687823	KF687845	KF687724	KF687867
*Colletotrichum subvariabile*	NN040649^*^	unknown	China	MZ664181	MZ799343	MZ664054	MZ673903	MZ595883	MZ674001
*Colletotrichum variabile*	NN040656^*^	unknown	China	MZ664182	MZ799344	MZ664055	MZ673904	MZ595884	MZ674002
*Colletotrichum vietnamense*	CBS 125477	*Coffea* sp.	Vietnam	KF687791	KF687768	KF687831	KF687854	KF687720	KF687876
	CBS 125478^*^	*Coffea* sp.	Vietnam	KF687792	KF687769	KF687832	KF687855	KF687721	KF687877
*Colletotrichum zhaoqingense*	NN058985^*^	dead petiole of palm	China	MZ664203	MZ799304	MZ664065	MZ673925	MZ595905	MZ674023
	NN071035	dead petiole of palm	China	MZ664204	MZ799305	MZ664066	MZ673926	MZ595906	MZ674024
	NN071036	dead petiole of palm	China	MZ664205	MZ799306	MZ664067	MZ673927	MZ595907	MZ674025

**Note**. “NA” indicates unavailable sequences; sequences produced in the current study are in bold, and * means ex-type strains.

First, BLAST searches of the ITS sequences preliminarily indicated that the four strains in this study belonged to the *Colletotrichum gigasporum* species complex. Subsequently, the reference sequences were downloaded from NCBI GenBank (https://www.ncbi.nlm.nih.gov/genbank/). All sequences were initially aligned with MAFFT v. (http://mafft.cbrc.jp/alignment/server/index.html) and manually edited in MEGA v. 7.0 (Kumar et al. 2016; Katoh et al. 2019). For phylogenetic analyses, the concatenated alignments of ACT, CHS-1, GAPDH, HIS3, ITS, and TUB2 sequences were analyzed using maximum likelihood (ML) and Bayesian inference (BI). ML and BI analyses were performed using RAxML-NG v. 1.1.0 and MrBayes v. 3.2.7a, respectively, with 64 threads on Linux (Miller et al. 2012; Ronquist et al. 2012; Stamatakis 2014). The best evolutionary model for each partition was determined using PartitionFinder 2 (Lanfear et al. 2017) with the AICc criterion and a greedy search algorithm and incorporated into the analyses. For the ML analyses, 1000 rapid bootstrap replicates were performed using the GTR+I+G model as the default. For the BI analyses, MrBayes was run with the stoprule=yes option (threshold ASDSF = 0.01). Convergence was further confirmed by inspecting the potential scale reduction factor (PSRF ≈ 1.0 for all parameters) and effective sample sizes > 200. The resulting trees were visualized in FigTree v. 1.4.4 (Rambaut 2018), and the final layout of the trees was edited in Adobe Illustrator CC 2019 (Adobe Systems, California, USA).

## Results

### Phylogenetic analyses

**Figure 1.**
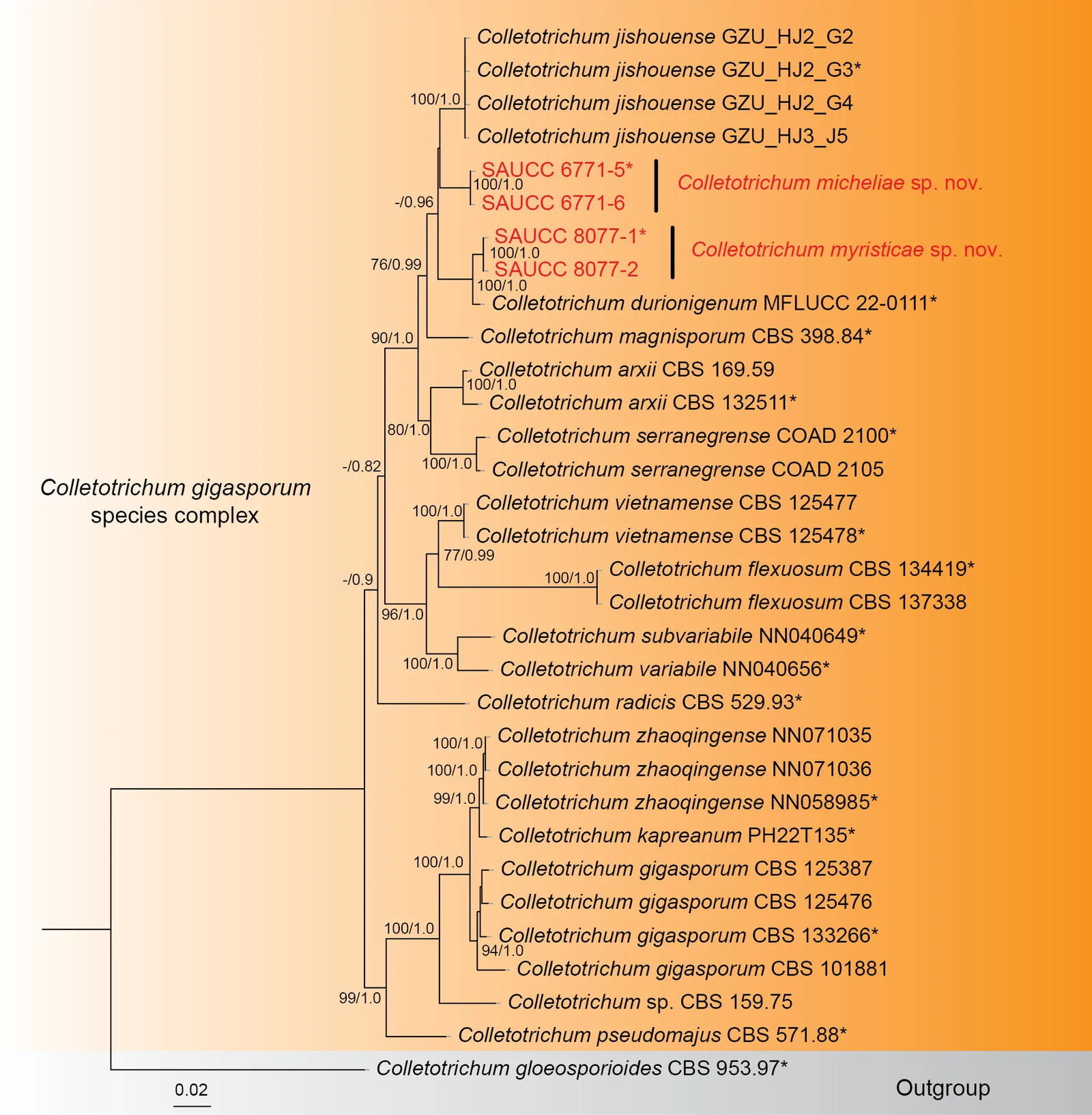
Maximum likelihood tree of the *Colletotrichum gigasporum* species complex generated from combined ACT, CHS-1, GAPDH, HIS3, ITS, and TUB2 sequence data. Bootstrap support values (BSV) ≥ 70% and Bayesian posterior probabilities (BPP) ≥ 0.80 are shown at the branches. Isolates from the present study are indicated in red, and * indicates ex-type strains.

To determine the phylogenetic position of these strains, combined ML and BI analyses of the ACT, CHS-1, GAPDH, HIS3, ITS, and TUB2 sequence data were conducted to construct phylogenetic trees. The phylogenetic analysis included 32 strains, with *Colletotrichum gloeosporioides* (CBS 953.97) serving as the outgroup. The final alignment comprised 2,466 concatenated characters, *viz*. 1–255 (ACT), 256–518 (CHS-1), 519–802 (GAPDH), 803–1183 (HIS3), 1184–1734 (ITS), and 1735–2466 (TUB2). The ML optimization likelihood was −10,158.175175. The matrix exhibited 879 distinct alignment patterns, with 18.34% of characters or gaps remaining undetermined. For BI, the alignment showed 885 unique site patterns (ACT and TUB2: 412, CHS-1: 66, GAPDH: 181, HIS3: 79, and ITS: 147). The GTR+G model for ACT and TUB2, the SYM+I+G model for CHS-1, the HKY+I model for GAPDH, the GTR+G model for HIS3, and the SYM+G model for ITS were selected and incorporated into the analyses. The Markov chain Monte Carlo (MCMC) analysis of the sequence data was performed over 5,000,000 generations, yielding 10,002 trees. After discarding 2,500 trees during the burn-in phase, the remaining trees were used to calculate posterior probabilities in the consensus trees. The topology of the ML tree concurred with that derived from BI; thus, only the ML tree is presented (Fig. 1). The strains SAUCC 6771-5 and SAUCC 6771-6 formed a fully supported lineage. Similarly, the strains SAUCC 8077-1 and SAUCC 8077-2 also formed a fully supported lineage. Based on morphological characteristics and molecular phylogenetic analyses, the four strains in this study were described as two new species.

### Taxonomy

#### Colletotrichum micheliae

Taxon classificationFungiGlomerellalesGlomerellaceae

J.W. Xia, Z.X. Dong & X.G. Zhang sp. nov.

9BC35A29-10DA-5A61-8881-73FED18ED966

863965

Fig. 2

##### Etymology.

Referring to the genus name of the host plant *Michelia × alba*.

**Figure 2.**
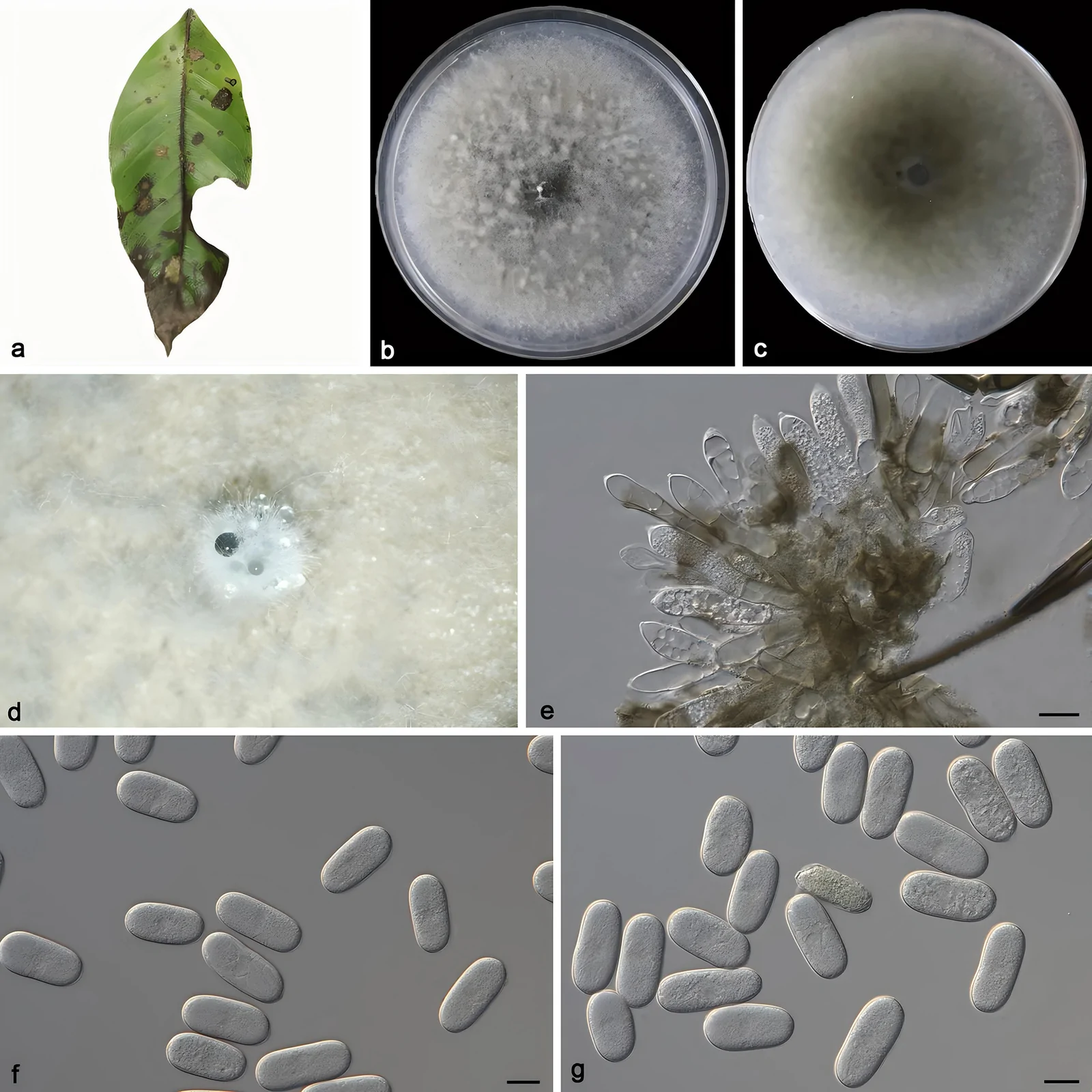
Morphology of *Colletotrichum micheliae*. **a**. Diseased leaf of *Michelia × alba*; **b, c**. Colony on PDA after 14 days at 25 °C (left: above, right: reverse); **d**. Immature conidiomata; **e**. Conidiogenous cells; **f, g**. Conidia. Scale bars: 10 µm (**e–g**).

##### Description.

Sexual morph: Not observed. Asexual morph: Vegetative hyphae hyaline to pale brown, smooth-walled, septate, branched. Conidiomata acervular, solitary or gregarious, subglobose, superficial to semi-immersed, grey to greyish brown. Appressoria and setae were not observed on PDA. Conidiophores hyaline to brownish, smooth-walled, septate, unbranched, cylindrical. Conidiogenous cells are hyaline, cylindrical, 16.5–30.5 × 6–8 µm (mean ± SD = 26.2 ± 4.7 × 7.1 ± 0.7 µm; *n* = 20), apex acute. Conidia are cylindrical, hyaline, with both ends rounded, smooth-walled and containing granular contents, 21.5–32 × 10–14 µm (mean ± SD = 26.3 ± 2.6 × 12.3 ± 1 µm; *n* = 50), with an *L/W* ratio of 2.1.

##### Culture characteristics.

Colonies on PDA incubated at 25 °C had an average radial growth rate of 10–16.5 mm/d, reached a diameter of 5 cm within 3 days, and covered a 9-cm Petri plate within 7 days. Surface white to gray-black, exhibiting a fluffy to cottony texture. Reverse gray to gray-black.

##### Materials examined.

China • Hainan Province, Danzhou City, near Hainan Tropical Botanical Garden, 19°30'42.98"N, 109°30'3.02"E, from a diseased leaf of *Michelia × alba*, 30 Oct. 2023, D.H. Li & Z.X. Dong, holotype HSAUP 6771-5, ex-holotype culture CGMCC 3.28944 = SAUCC 6771-5 • *Ibid*., HSAUP 6771-6, living culture SAUCC 6771-6.

##### Notes.

*Colletotrichum micheliae*, collected from *Michelia × alba* in Hainan Province, forms a fully supported clade (BSV = 100%, BPP = 1.0) sister to *C. jishouense* (from *Nothapodytes pittosporoides*), *C. myristicae* (from *Myristica fragrans*), and *C. durionigenum* (from rotting durian fruit). The pairwise DNA sequence dissimilarities between *C. micheliae* and *C. jishouense* were 2.07% in ACT (5/242 base pairs [bp], no gaps), 6.85% in GAPDH (5/73 bp, no gaps), 1.38% in ITS (7/506 bp, 4 gaps), and 3.30% in TUB2 (15/455 bp, 2 gaps). Because the CHS-1 and HIS3 sequences of *C. jishouense* are unavailable, sequence comparison between *C. micheliae* and *C. jishouense* is thus limited. The pairwise DNA sequence dissimilarities between *C. micheliae* and *C. myristicae* were 2.89% in ACT (7/242 bp, no gaps), 1.90% in CHS-1 (5/263 bp, no gaps), 8.00% in GAPDH (8/100 bp, no gaps), 2.67% in HIS3 (10/375 bp, no gaps), 2.24% in ITS (12/536 bp, 3 gaps), and 5.15% in TUB2 (37/718 bp, 16 gaps). The pairwise DNA sequence dissimilarities between *C. micheliae* and *C. durionigenum* were 1.93% in ACT (4/207 bp, no gaps), 2.51% in CHS-1 (6/239 bp, no gaps), 11.11% in GAPDH (8/72 bp, no gaps), 2.17% in ITS (11/507 bp, 5 gaps), and 3.48% in TUB2 (16/460 bp, 2 gaps). Because the HIS3 sequence of *C. durionigenum* is unavailable, sequence comparison between *C. micheliae* and *C. durionigenum* is thus limited. Moreover, *C. micheliae* differs from *C. jishouense* in its larger conidiogenous cells (16.5–30.5 × 6–8 µm vs. 4–11 × 2–3 µm) and conidia (21.5–32 × 10–14 µm vs. 5–14 × 3–5 μm) (Zhou et al. 2019). *C. micheliae* differs from *C. myristicae* and *C. durionigenum* in its shorter conidia (21.5–32 µm vs. 30–38.5 µm vs. 29–40 µm) (Armand et al. 2023).

#### Colletotrichum myristicae

Taxon classificationFungiGlomerellalesGlomerellaceae

J.W. Xia, Z.X. Dong & X.G. Zhang sp. nov.

CA7250F1-1F3C-5943-AA31-5E1A9139E585

863966

Fig. 3

##### Etymology.

Referring to the genus name of the host plant *Myristica fragrans*.

**Figure 3.**
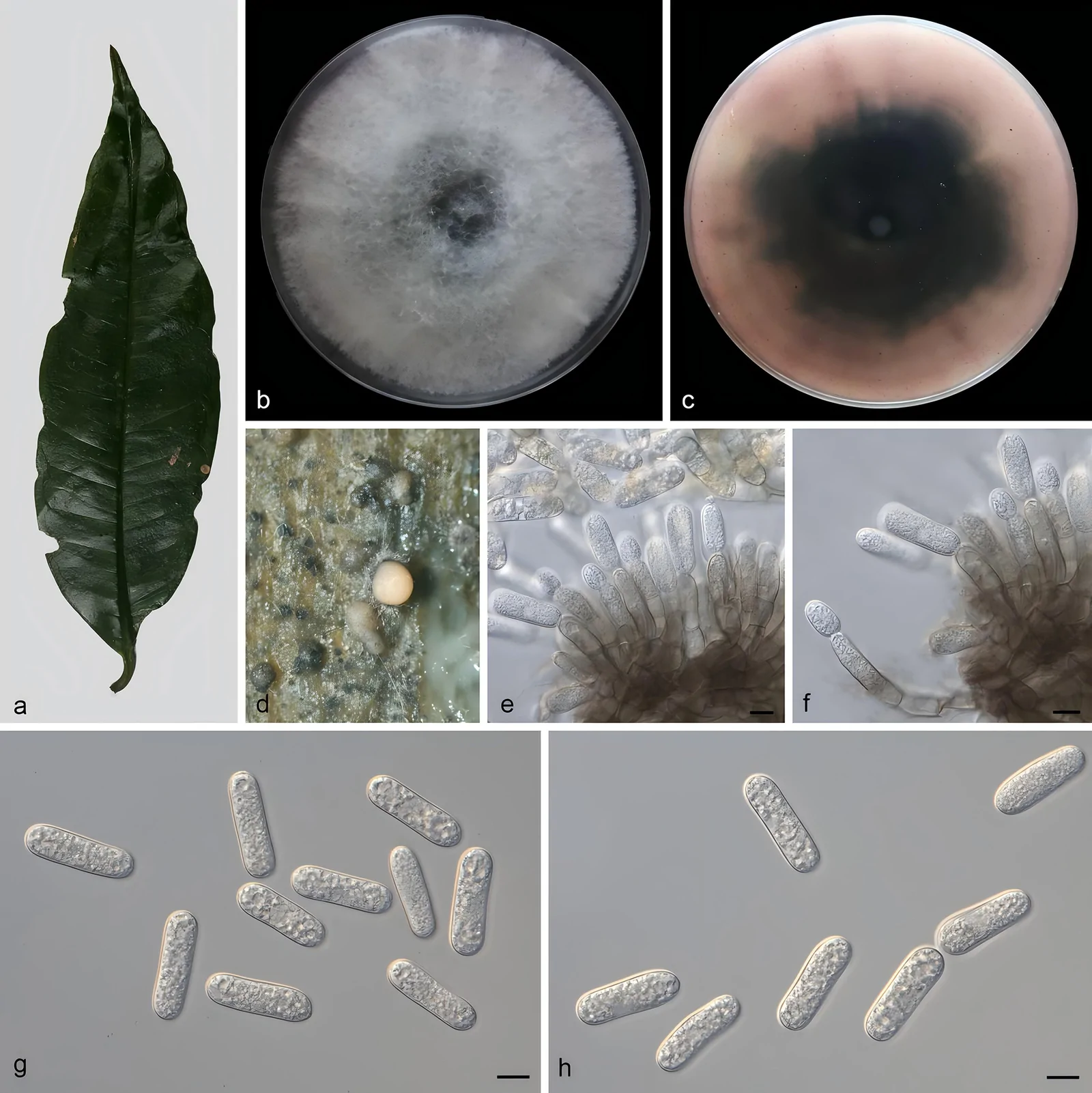
Morphology of *Colletotrichum myristicae*. **a**. Diseased leaf of *Myristica fragrans*; **b, c**. Colony on PDA after 14 days at 25 °C (left: above, right: reverse); **d**. Conidiomata; **e, f**. Conidiogenous cells and conidia; **g, h**. Conidia. Scale bars: 10 µm (**e–g**).

##### Description.

Sexual morph: Not observed. Asexual morph: Vegetative hyphae hyaline to pale brown, smooth-walled, septate, branched. Conidiomata acervular, solitary or gregarious, subglobose, superficial to semi-immersed, milk white to yelloish. Appressoria and setae were not observed on PDA. Conidiophores hyaline to brownish, smooth-walled, septate, branched, cylindrical. Conidiogenous cells are hyaline, cylindrical, 15.5–33 × 7.5–9.5 µm (mean ± SD = 22.7 ± 5.6 × 8.3 ± 0.5 µm; *n* = 20), apex rounded. Conidia are cylindrical, hyaline, with both ends rounded, smooth-walled and containing granular contents, 30–38.5 × 9.5–12.5 µm (mean ± SD = 33.8 ± 1.8 × 10.8 ± 0.8 µm; *n* = 50), with an *L/W* ratio of 3.1.

##### Culture characteristics.

Colonies on PDA incubated at 25 °C had an average radial growth rate of 10–16.5 mm/d, reached a diameter of 5 cm within 3 days, and covered a 9-cm Petri plate within 7 days. Surface pale pink to dark gray, exhibiting a fluffy to cottony texture. Reverse pale pink to black.

**Materials examined**. China • Hainan Province, Wuzhishan City, 18°54'20.33"N, 109°40'59.99"E, from a diseased leaf of *Myristica fragrans*, 28 Mar. 2024, Z.X. Dong, holotype HSAUP 8077-1, ex-holotype culture CGMCC 3.28945 = SAUCC 8077-1 • *Ibid*., HSAUP 8077-2, living culture SAUCC 8077-2.

##### Notes.

*Colletotrichum myristicae*, collected from *Myristica fragrans* in Hainan Province, forms a fully supported clade (BSV = 100%, BPP = 1.0) sister to *C. jishouense* (from *Nothapodytes pittosporoides*), *C. micheliae* (from *Michelia × alba*), and *C. durionigenum* (from rotting durian fruit). The pairwise dissimilarities of DNA sequences between *C. myristicae* and *C. jishouense* were 2.48% in ACT (6/242 bp, no gaps), 14.08% in GAPDH (10/71 bp, no gaps), 1.40% in ITS (7/501 bp, 1 gap), and 3.52% in TUB2 (16/455 bp, no gaps). As the DNA sequences of CHS-1 and HIS3 in *C. jishouense* are unavailable, sequence comparison between *C. myristicae* and *C. jishouense* is thus limited. The differences between *C. myristicae* and *C. micheliae* are mentioned in the notes for *C. micheliae*. The pairwise dissimilarities of DNA sequences between *C. myristicae* and *C. durionigenum* were 1.00% in ACT (2/207 bp, no gaps), 1.26% in CHS-1 (3/239 bp, no gaps), 0.79% in ITS (4/504 bp, 2 gaps), 0.87% in TUB2 (4/460 bp, no gaps), and no difference in GAPDH (0/72 bp, no gaps). As the DNA sequence of HIS3 in *C. durionigenum* is unavailable, sequence comparison between *C. myristicae* and *C. durionigenum* is thus limited. Moreover, *C. myristicae* differs from *C. jishouense* in its larger conidiogenous cells (15.5–33 × 7.5–9.5 µm vs. 4–11 × 2–3 µm) and conidia (30–38.5 × 9.5–12.5 µm vs. 5–14 × 3–5 µm) (Zhou et al. 2019). *C. myristicae* differs from *C. durionigenum* by producing shorter conidiogenous cells (15.5–33 µm vs. 18.5–39 µm). The conidia of *C. myristicae* are slightly wider than those of *C. durionigenum* (9.5–12.5 µm vs. 8.5–11.5 µm). Additionally, *C. durionigenum* (MFLU 22-0194) produces conidia with pale purple pigments that have not been found in *C. myristicae* (Armand et al. 2023).

## Discussion

Despite the instability of morphological traits under varying growth conditions, species within the *Colletotrichum gigasporum* species complex produce larger conidia than most other *Colletotrichum* species, which serves as a valuable diagnostic feature at the species complex level (Liu et al. 2014). Moreover, single-gene data, such as ITS, usually lack the resolving power needed for species-level identification across most *Colletotrichum* species complexes, and multilocus phylogenetic analyses are essential for the formal description of species in the genus *Colletotrichum* (Hyde et al. 2009; Cannon et al. 2012; Liu et al. 2014; Jeewon and Hyde 2016).

The *Colletotrichum gigasporum* species complex seems to be widely distributed geographically, and its species have been found in Asia (China, India, Japan, Korea, the Philippines, Thailand, and Vietnam), Africa (East Africa and Madagascar), Central and South America (Brazil, Chile, Colombia, Ecuador, Guyana, Mexico, and Panama), and Oceania (New Zealand) (Liu et al. 2014, 2022; Silva et al. 2018; Zhou et al. 2019; Jayawardena et al. 2022; Armand et al. 2023; Takata et al. 2024). According to previous studies, species of the *Colletotrichum gigasporum* species complex have been recorded as plant pathogens and endophytes, as well as from air and stored grain (Liu et al. 2014, 2022; Silva et al. 2018; Zhou et al. 2019; Jayawardena et al. 2022; Armand et al. 2023; Takata et al. 2024).

Previous researchers have recorded *C. arecicola*, *C. brevisporum*, *C. cordylinicola*, *C. endophyticum*, *C. fructicola*, *C. gigasporum*, *C. gloeosporioides*, *C. karstii*, *C. ledongense*, *C. musicola*, *C. plurivorum*, *C. queenslandicum*, *C. siamense*, *C. tropicale*, and *C. wanningense* from areca palm, coffee, rubber, and tea-oil camellia in Hainan Province, China (Cao et al. 2019a, 2019b, 2020; Zhu and He 2023; Xu et al. 2025). *Colletotrichum magnoliae* and *C. siamense* were recorded from *Michelia × alba* in China (Tai 1979; Qin et al. 2021). *Colletotrichum gloeosporioides* was recorded from *Myristica fragrans* in India and Indonesia (Mathur 1979; Farr et al. 2026). In the present study, two new *Colletotrichum* species, *C. micheliae* and *C. myristicae*, are described from *Michelia × alba* and *Myristica fragrans* in Hainan Province, China. These findings enhance our understanding of the biodiversity of the genus *Colletotrichum* in Hainan Province and contribute valuable data for future research on fungal systematics and biodiversity conservation. However, although *C. micheliae* and *C. myristicae* were isolated from diseased plant leaves, further research is needed to verify through Koch’s postulates whether they are pathogens.

## Supplementary Material

XML Treatment for Colletotrichum micheliae

XML Treatment for Colletotrichum myristicae

## Additional information

### Artificial Intelligence (AI) use

The authors accept full responsibility for the content of the manuscript, including the disclosure of any use of AI.

No AI tools were used in the preparation of this manuscript.

### Author ORCIDs

Z. Dong https://orcid.org/0009-0002-3031-8151

Y. Yuan https://orcid.org/0009-0005-8799-3950

Z. Zhang https://orcid.org/0000-0002-4824-9716

X. Zhang https://orcid.org/0000-0001-9733-8494

J. Xia https://orcid.org/0000-0002-7436-7249

### Data availability

All of the data that support the findings of this study are available in the main text.
